# Maternal resistin predisposes offspring to hypothalamic inflammation and body weight gain

**DOI:** 10.1371/journal.pone.0213267

**Published:** 2019-03-07

**Authors:** Ghislaine Poizat, Coralie Alexandre, Sarah Al Rifai, Laure Riffault, Delphine Crepin, Yacir Benomar, Mohammed Taouis

**Affiliations:** CNRS NeuroPSI UMR 9197, Molecular Neuroendocrinology of Food Intake, University Paris-Sud, University Paris-Saclay, Orsay, France; East Tennessee State University, UNITED STATES

## Abstract

Resistin promotes hypothalamic neuroinflammation and insulin resistance through Toll like receptor 4 (TLR4), this hormone is thought to be a link between obesity and insulin-resistance. Indeed, resistin plasma levels are higher in obese and insulin resistant subjects. However, the impact of maternal resistin on the predisposition of offspring to hypothalamic neuroinflammation is unknown. Here, female mice were treated with resistin during gestation/lactation periods, then hypothalamic neuroinflammation was investigated in male offspring at p28 and p90. At p28, resistin increased the expression of inflammation markers (IL6, TNFα and NFκB) and TLR4 in the hypothalamus and decreased both hypothalamic insulin and leptin receptors’ expression. The hypothalamic up-regulation IL6, TNFα and TLR4 was sustained until p90 promoting most likely hypothalamic inflammation. Maternal resistin also increased IL6 and TNFα in the adipose tissue of offspring at p90 associated with a higher body weight gain. In contrast, liver and muscle were not affected. These findings reveal that the augmentation of maternal resistin during gestation and lactation promotes hypothalamic and adipose tissue inflammation of offspring as evidenced by sustained increase of inflammation markers from weaning to adulthood. Thus, maternal resistin programs offspring hypothalamic and adipose tissue inflammation predisposing then offspring to body weight gain.

## Introduction

The low-grade inflammation in obese, insulin-resistant or type-2 diabetes subjects is an important issue in developmental programming of inflammation and its long-term consequences on offspring [1, 2]. Indeed, hypercaloric maternal diet programs inflammation in offspring tissues [3–6]. Moreover, accumulated evidences suggest that changes of inflammatory markers in early life could have a strong impact on the prevalence of metabolic and cardiovascular diseases [7–9]. It has been suggested that offspring inflammation is promoted by LPS-induced maternal inflammation. This treatment led to strong changes in offspring immune system evidenced by more pro-inflammatory macrophages M1 and a higher Interleukin-1β (IL1β) production [10, 11]. It is also well documented that maternal obesity increased pro-inflammatory cytokine in the placenta enhancing then inflammatory responses of offspring. Furthermore, fatty acids, cholesterol, and triglyceride plasma levels were increased in fetuses born to obese ewes that also exhibited up-regulated Toll Like Receptor 4 (TLR4), Nuclear Factor-kappa B (NFκB) and c-Jun N-Terminal kinase (JNK) [12]. TLR4 is considered as the binding site for LPS and, more recently TLR4 was described as a receptor for resistin, an adipokine [13, 14]. Resistin is implicated in the onset of low-grade inflammation found in obese or insulin resistant rodent models [15, 16]. Indeed, resistin decreases insulin responsiveness and contributes to the onset of type 2 diabetes [17]. Interestingly, intracerebroventricular (ICV) resistin treatment induces overall inflammation and insulin resistance through the activation of hypothalamic TLR4 signaling pathway. This treatment also altered both adiponectin and FGF21 signaling pathways known as insulin sensitizers [18]. These findings are in line with previous studies suggesting that resistin links obesity to insulin resistance [17]. The impact of resistin during pregnancy on the placenta and offspring has been suspected. Indeed, resistin is expressed in human placenta, and it has been suggested that placental resistin modulates insulin sensitivity during pregnancy [19]. Furthermore, resistin plasma levels are increased in women with gestational diabetes [20–22]. However, this augmentation remains controversial, since other studies reported that gestational diabetes does not affect plasma resistin levels [23]. Moreover, several studies reported high resistin plasma levels in pregnant women as compared to non-pregnant women [22, 24]. It has been also reported that TLR4 was highly expressed in the placenta of obese women as compared to lean women, suggesting the implication of TLR4 in placental inflammation that could affect the fetus [25]. Based on our previous findings demonstrating the implication of hypothalamic resistin/TLR4 signaling pathway in the onset of inflammation and insulin resistance [14], we hypothesized that maternal resistin could be implicated in the predisposition of offspring to inflammation and insulin resistance especially when subjected to inappropriate diet such as HFD. Indeed, the role and the implication of maternal resistin on the development of inflammation in offspring are still unknown especially at the hypothalamic level. Thus, in the present study, we aim to investigate whether maternal resistin has long-term effects on the predisposition of offspring to develop inflammation and metabolic disorders when subjected to HFD, with a special focus on hypothalamic neuroinflammation, termed as perinatal programmed hypothalamic inflammation. Indeed, HFD is known to promote inflammation and in this study feeding CC and CR offspring with HFD from p60 to p90 will respond to the question whether offspring born to dams treated with resistin (CR group) are prone to hypothalamic inflammation and alterations of metabolic parameters as compared CC group. For this purpose, pregnant mice were treated with resistin during gestation and lactation and then we analyzed the expression of inflammation markers in the hypothalamus and adipose tissue together with body weight gain and metabolic parameters of male offspring at p28 and adult mice following a 30 days HFD challenge.

## Methods

### Animals

**Fig 1:**
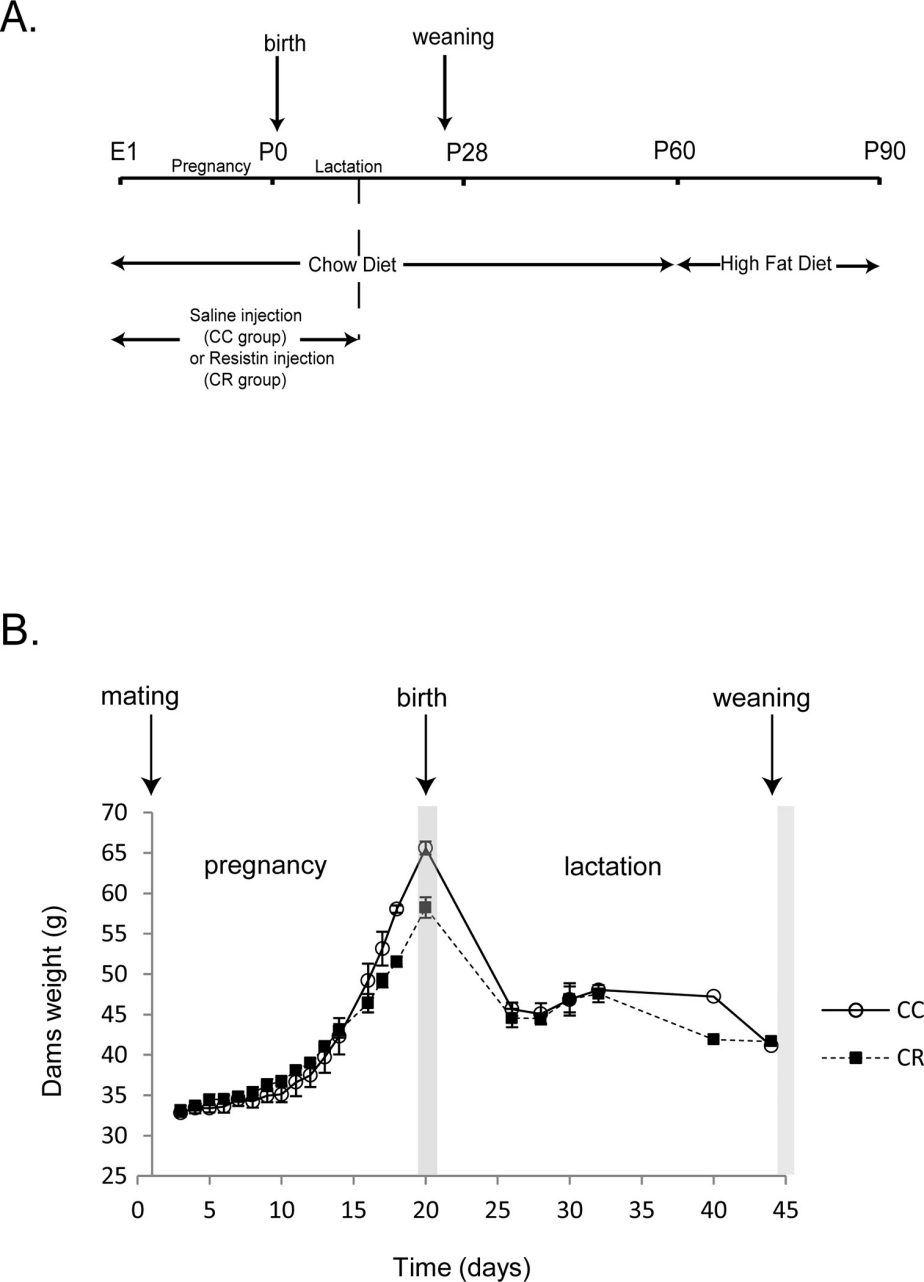
Impact of resistin treatment on dams’ body weight during pregnancy and lactation. Experimental protocol: Dams were treated with saline (CC group) or resistin 20 μg/animal (CR group) and fed with standard diet (Chow Diet). Dams received daily injections of saline or resistin during pregnancy and every two days during the first two weeks of lactation period. From weaning to p60, offspring were fed Chow diet and then switched to high fat diet (HFD) from p60 to p90. Sacrifice of animals has been done just after weaning for dams and a part of offspring, and at p90 for the rest of offspring. B- Dams body weight from pregnancy to weaning. Dams body weight (g) is expressed as mean ± SEM. No significant difference between CC and CR Group (n = 4) has been noticed using non-parametric ANOVA statistical test for longitudinal data.

Swiss adult female mice (Janvier labs) were randomly divided in 2 groups in individual cages, in a temperature-controlled environment with 12-h light 12-h dark cycles and unrestricted access to water and standard chow diet (SAFE, Augy, France). Housing females were mated with Swiss CD1 males during two days. All experimental procedures were performed according to the institutional guidelines of laboratory animal care and approved by the governmental commission of animal research: Ethic Committee for animal experimentation of Paris Center and South # 59 (France), with authorization # 2016–3. The number of animals used in each group has been determined after power test analysis using R Software Two Sample t-test calculation (RcmdrPlugin.IPSUR) as previously described [26]. Pregnant females were maintained in individual cages and during pregnancy; dams were daily injected intraperitoneally with physiologic saline (CC for **C**how Diet **C**ontrol group) or with human Resistin (obtained from Shenandoah Biotechnology, Warwick, PA, USA) at a dose of 20μg/animal (CR for **C**how diet **R**esistin group) (Fig 1A). Body weight of dams was measured every day during gestation and every 2–3 days during lactation period until weaning. During the first 2 weeks of lactation period, dams were injected intraperitoneally with physiologic saline (CC group) or with human Resistin (CR group) at a dose of 20μg/animal every two days. Litter size was adjusted to 13–14 animals per dam in average. At weaning (p28), only males were used for the following experiments. Indeed, in this pilot project in the field of maternal programmed neuroinflammation we firstly focused on male in order to avoid potential sexual dimorphism for the studied parameters. Additionally, for budgetary reasons we decided to perform another investigation for female. Thus, female mice were donated to colleagues for another project. For the present experiment, 10 males per group were maintained and fed standard chow diet (19.6% proteins, 11.9% lipids, 68.5% carbohydrates) until day 60 (p60). At this age, animals were switched to a high fat diet (HFD 235HF-U8955 version2-SAFE, 15.6% proteins, 44.6% lipids, 39.9% carbohydrates, these values are percent of total calories) from p60 to p90. Animals weight and food intake are regularly measured. Indeed, offspring body weight and food intake were measured twice a week from P60 to P90.

Sacrifice of overnight fasted animals was done at weaning for dams and p28 or p90 for offspring.

### Serum and tissue collection

Mice were rapidly decapitated and then blood was collected immediately in tubes containing 12U/mL of heparin, plasma was prepared after blood centrifugation at 2000 g at 4°C during 15 minutes and then stored at -20°C until assayed for ELISA experiments. Brain was extracted and the hypothalamus was immediately dissected out, fiash-frozen in liquid nitrogen and then store at -80°C. The quality of hypothalamus dissection is routinely validated by the measurement of Pro-Opiomelanocortin (POMC) expression. Liver, muscle and abdominal adipose tissue (AAT) were also collected and flash-frozen in liquid nitrogen and then stored at -80°C.

### Measurement of plasma hormones and cytokines

Plasma levels of resistin, insulin, leptin, Interleukin-6 (IL6) and Tumor Necrosis Factor (TNFα were determined by ELISA. ELISA kits for IL6 (M6000B) and TNFα and resistin (MRSN00) were purchased from R&D Systems (Minneapolis, MN, USA) and those for insulin (EZRMI-13K) and leptin (EZML-82K) were from Millipore (Burlington, VT, USA). The measurements were performed according to manufacturer recommendations using plasma samples stored at -20°C.

### Glucose tolerance test (GTT)

The intraperitoneal GTT was performed after fasting the mice overnight. Basal glycemia (time 0) of overnight fasted mice (p28 or p90) is measured prior to intraperitoneal injection of glucose (2 mg/kg) and then tail blood samples were collected to measure glycemia 15, 30, 60, 120 min and 200 min after the injection. Plasma glucose was monitored using Accu-Check system (Roche, France). The first drop of blood is discarded and the second used for measurement.

### Hormones and chemical

Human Resistin is from Shenandoah Biotechnology (Warwick, PA, USA). D-Glucose is from Euromedex (Souffelweyersheim, France).

### RNA extraction and quantitative RT-PCR

Total RNA from mice was isolated using Trizol reagent (Life Technologies, Carlsbad, CA, USA). 0.5 μg of RNA was reverse transcribed and the cDNA were submitted to Quantitative real-time PCR analysis using SYBR Green QPCR system (Biorad, Les Ulis, France). qScript cDNA Synthesis Kit and SYBR Green mix kit are from VWR (Fontenay sous Bois, France). Specific primers are from Life Technologies (Carlsbad, CA, USA): IL6 (sense: 5’ GCCTTCTTGGGACTGATGCTGGT3’, antisense: 5’ TCCTCTGTGAAGTCTCCTCTCCG3’), TNFα (sense: 5’AAGTCAACCTCTCTGCC3’, antisense: 5’ TCCAAAGTAGACCTGCCCG3’); TLR4 (sense: 5’CTGGGGAGGCACATCTTCTGG3’, antisense: 5’ TGCCGTTTCTTGTTCTTCCTCTGCT3’), resistin (sense: 5’CCCAGAAGGCACAGCAG3’, antisense: 5’TCACGAATGTCCCACGAGC3’), NFκb (sense: sense: 5’ ACAGATGGGCTACACAGAGG3’, antisense: 5’ GTGGAGGAAGACGAGAGAGG3’), GFAP (sense: 5’AAGAGACAGAGGAGTGGTATCGG3’, antisense: 5’ GCTTCGTGCTTGGCTTGGC3’), IBA1 (sense: 5’ GAAAGGGGAAGTGTGAG A3’, antisense: 5’ TGGCTCATGGCTCCTCA G3’), CD68 (sense: 5’ GGGGCTCTTGGGAACTACA3’, antisense: 5’ GAGAGAGACAGGTGGGGATG3’), ICAM1 (sense: 5’ GACACCCCTGACCTCCTG3’, antisense: 5’ CTGCTGGTTTGTGCTCTCCTG3’), IR (sense: 5’ GCCGCTCCTATGCTCTGGTATC3’, antisense: 5’ GAGTGATGGTGAGGTTGTGTTTGC3’), PTP1B (sense: 5’ CTCTTCTCCAGCCACCAGT3’, antisense: 5’ CCACCATCCGTCTCCTAACT3’), SOCS3 (sense: 5’ CCAGCTCCAGCTTCTTTCAAGTG3’, antisense: 5’ GAGAGTCCGCTTGTCAAAGGTATTG3’), LepR (Taqman primers designed by Applied Biosystems). All results are normalized with 18S and GAPDH mRNA expression (and sometimes Actin mRNA). These house-keeping genes were previously tested in this model and specifically in each tissue. All samples were measured in duplicates.

### Data analysis and statistics

Dams weight and plasma glucose levels during GTT are expressed as mean ±SEM. GTT Area under curve quantification has been done with imageJ and Excel Software. Metabolic/endocrine data and qPCR mRNA results are represented as boxplots made with R software, showing median and interquartile range (Q1-Q3).

The number of animals used in each experiment was determined by power test analysis using R Software Two sample t-test calculation (RcmdrPlugin.IPSUR) based on previous experiments (14, 18) considering an α risk of 0,05% and a power of 90% (p<0.05).

Mann & Whitney non-parametric test has been used for all statistical analysis using Excel or R software (n = 4–9 per group). For dams’ body weight analysis and glucose tolerance test (GTT) experiments, non parametric ANOVA including longitudinal data has been performed withy R Software and nparLD library.

## Results

### Increased body weight, insulinemia and leptinemia of offspring born to dams treated with resistin

Female mice were treated with resistin or placebo during a period covering from pregnancy to the two first weeks of lactation (Fig 1A). Firstly, we have measured dams’ body weight from pregnancy to weaning. We noticed that resistin treatment did not affect body weight (Fig 1B). Moreover, this treatment did not alter metabolic or endocrine parameters of dams despite a slight but non-significant increase of leptin plasma levels (Table 1). The offspring were subjected to normal diet from weaning (p28) to p60 and then switched to high fat diet from p60 to p90. At p28, offspring exhibit a slight but not significant increase of body weight (Table 1). Plasma glucose, leptin or insulin levels of offspring born to dams treated with resistin (CR) were not affected. At p90, body weight, insulinemia and leptinemia were significantly increased in CR as compared to CC group (Table 1).

**Table 1:** Impact of maternal resistin on metabolic and endocrine parameters.

	Dams (at weaning)		P28 Offspring		P90 Offspring	
	CC	CR	CC	CR	CC	CR
Body weight (g)	41.1±1	41.7±0.3	12.69±0.26	17.08±1.95	40.5±1.1	45.8±1.8*
Plasma Glucose (g/L)	1,74±0.06	1,64±0.12	0.96±0.08	0.84±0.1	0.88±0.04	0.86±0.07
Plasma Insulin (ng/mL)	0.72±0.04	0.5±0.22	0.52±0.14	0.48±0.09	0.2±0.02	1.16±0.41*
Plasma Leptin (ng/mL)	1.97±0.63	4.70±1.09	0.05±0.03	0.20±0.14	0.52±0.21	3.56±1.25*
Plasma Resistin (ng/mL)	42.54±3.57	45.41±0.60	12.56±1.01	6.47±0.61*	11.47±2.44	7.74±2.16

Impact of maternal resistin on Body weight and metabolic/endocrine parmeters. Dams were treated with physiologic saline (CC) or resistin (20 mg/animal) during pregnancy and lactation. Body weight, plasma levels of glucose, insulin, leptin and resistin were measured in dams at weaning, and offspring at p28 and p90. All data are expressed as mean ± SEM (n = 4–9 per group for body weight analysis and 4–5 per group for plasma measurements).

*P<0.05 compared to CC group by non-parametric Mann & Whitney statistical test.

Resistin plasma levels were not affected by dams’ resistin treatment. Indeed, a slight but non-significant increase was found in CR dams as compared to CC dams. In offspring at p90, resistin plasma levels were not affected for CC and CR, respectively. In contrast, in p28 offspring resistin plasma levels were significantly decreased in CR group as compared to CC (Table 1).

Moreover, the plasma levels of pro-inflammatory markers IL6 and TNFα were measured in dams and offspring. In dams plasma levels of IL6 (15.96 ± 5.95 pg/mL vs 18.91± 16 pg/mL for CC and CR, respectively) and TNFα (11.49 ± 3.25 pg/mL vs 11.81 ± 5.35 pg/mL for CC and CR, respectively) were not affected by resistin treatment. At p28, IL6 and TNFα plasma levels were not detectable (below ELISA KIT threshold sensitivity). Similarly to dams, in p90 offspring plasma levels of IL6 (4.77 ± 0.27 pg/mL vs 5.58 ±0.26 pg/mL for CC and CR, respectively) and TNFα(12.74± 5.94 pg/mL vs 12.32 ± 5.24 pg/mL for CC and CR, respectively) exhibit similar values when comparing CC to CR groups.

### Impaired glucose tolerance of adult offspring born to dams treated with resistin

**Fig 2:**
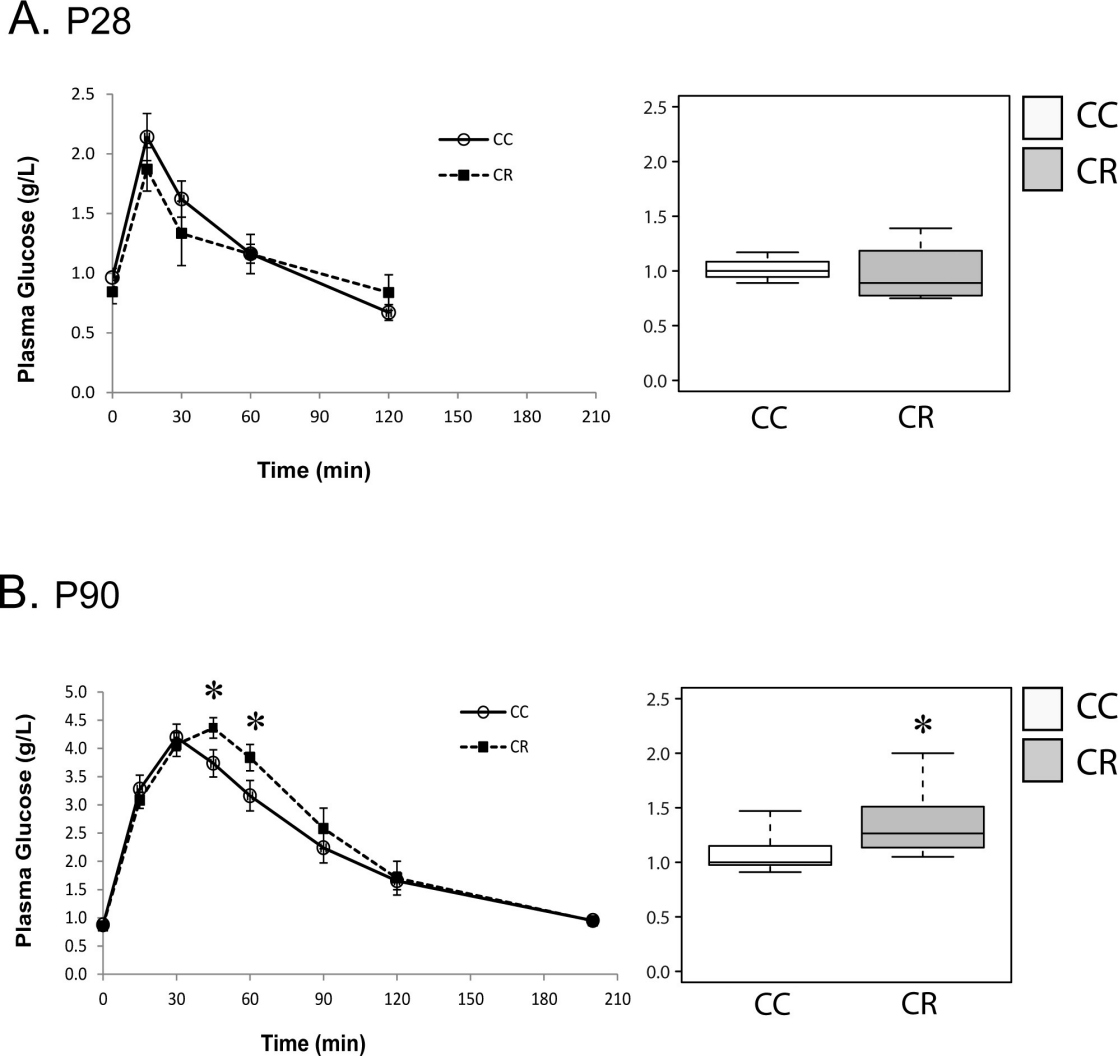
Maternal resistin treatment alters offspring glucose tolerance. Glucose Tolerance Tests were performed on p28 (A, n = 4) and p90 (B, n = 8), animals received through IP injection glucose (2mg/kg) and then blood glucose levels (g/L) were measured at the indicated times. Basal glycaemia was measured just before injection. The area under the curve representative of glucose tolerance was measured using ImageJ software. Data are presented as boxplots (right panels) and expressed as mean ± SEM in graphs. For the GTT analysis, the repeated measures were analyzed by non-parametric ANOVA including longitudinal data. The area under the curve (AUC) of GTT and the individual comparison of time course points were analyzed using on non-parametric Mann Whitney test. *P<0.05 when comparing CC group to CR group using non-parametric Mann & Whitney statistical test.

To determine the long-term effect of maternal resistin treatment on glucose tolerance of offspring, we have performed glucose tolerance test (GTT) at p28 and p90 in both CC and CR groups. At p28, GTT was similar in CC and CR groups and in both groups glycemia returned to basal levels within 120 min after glucose load (Fig 2A). At p90, GTT was performed in both CC and CR groups that were subjected to HFD between p60 and p90. The area under the curve (AUC) of GTT was significantly increased in CR as compared to CC group based on non-parametric Mann Whitney test. This indicates decreased glucose tolerance in CR group as compared to CC group (Fig 2B). For the GTT analysis, the repeated measures were analyzed by non-parametric ANOVA including longitudinal data, this analysis revealed a non-significant difference between CC and CR groups. However, considering each time course point individually, we show significant differences between CC and CR groups at 45 and 60 min. In both groups, glycemia returned to the basal level after 200 min after glucose load (Fig 2B).

### Increased gene expression of neuroinflammation and reactive gliosis markers in the hypothalamus of offspring born to dams treated with resistin

We have measured the expression levels of several markers of neuroinflammation and reactive gliosis that mirror the activation of microglia cells, as well as genes involved in the control of energy homeostasis in the hypothalamus of dams and offspring.

**Fig 3:**
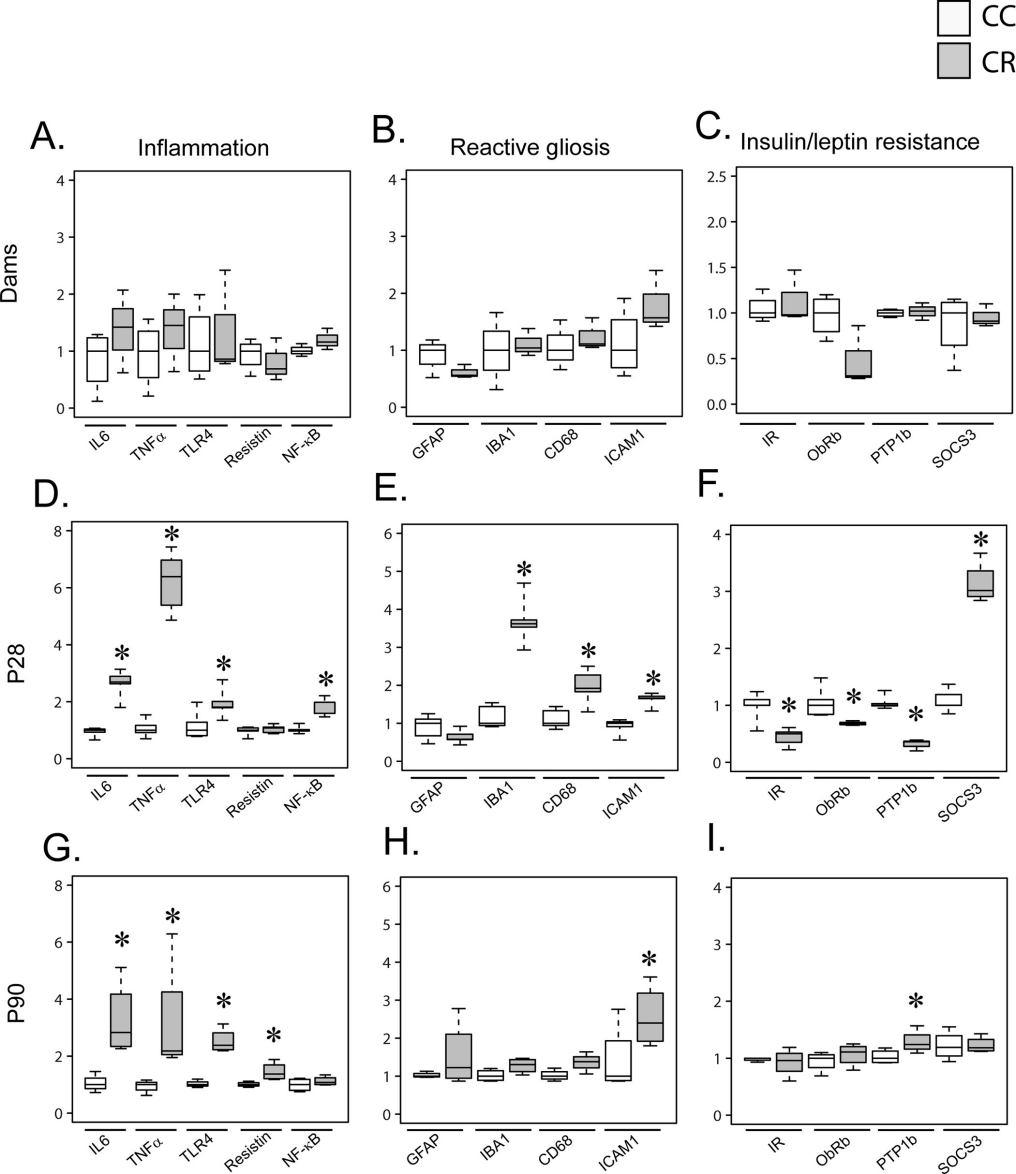
Maternal resistin increases offspring hypothalamic inflammation and reactive gliosis markers. Total mRNA prepared from the hypothalamus of dams and offspring (p28 and p90) were used to determine gene expression of different markers using RT-qPCR. Gene expression was normalized to GAPDH and 18S. Hypothalamic inflammatory markers are presented in panel A for dams, panel D for p28 offspring and panel G for p90 offspring. Hypothalamic gliosis markers are presented in panel B for dams, in panel E for p28 offspring and panel H for p90 offspring. Hypothalamic insulin/leptin responsiveness markers are presented in panel C for dams, panel F for p28 offspring and panel I for p90 offspring. Data are presented as boxplots (n = 4–5). *P<0.05 when comparing CC group to CR group using non-parametric Mann & Whitney statistical test.

In dams, resistin treatment did not affect hypothalamic expression of neuroinflammation and reactive gliosis markers as well as IR, ObRb, PTP1B and SOCS3 (Fig 3A, 3B and 3C).

In offspring, at p28, CR group exhibits a significantly higher expression of neuroinflammation markers as IL6, NFκb, TNFα and TLR4, the latest is known to be the binding site for LPS and resistin (Fig 3D). The expression levels of resistin were not affected. CR group also exhibits a higher expression of reactive gliosis markers such as CD68, IBA1 and ICAM1 as compared to CC group revealing the activation of microglia cells (Fig 3E). Additionally, IR and ObRb hypothalamic expression is diminished in CR as compared to CC group at p28 as well as PTP1-B expression (Fig 3F). In contrast, the expression of SOCS3, a negative regulator of both insulin and leptin signaling, is increased CR group as compared to CC group (Fig 3F).

At p90, the long-term impact of maternal resistin is evidenced by a significant increase of hypothalamic IL6, TNFα, TLR4 and resistin gene expression (Fig 3G). ICAM1 expression is also significantly increased in CR group, however the other measured gliosis markers were not affected (Fig 3H). We have also shown that PTP1B is significantly increased in CR group as compared to CC group without modification of IR, ObRb or SOCS3 expression (Fig 3I).

**Fig 4:**
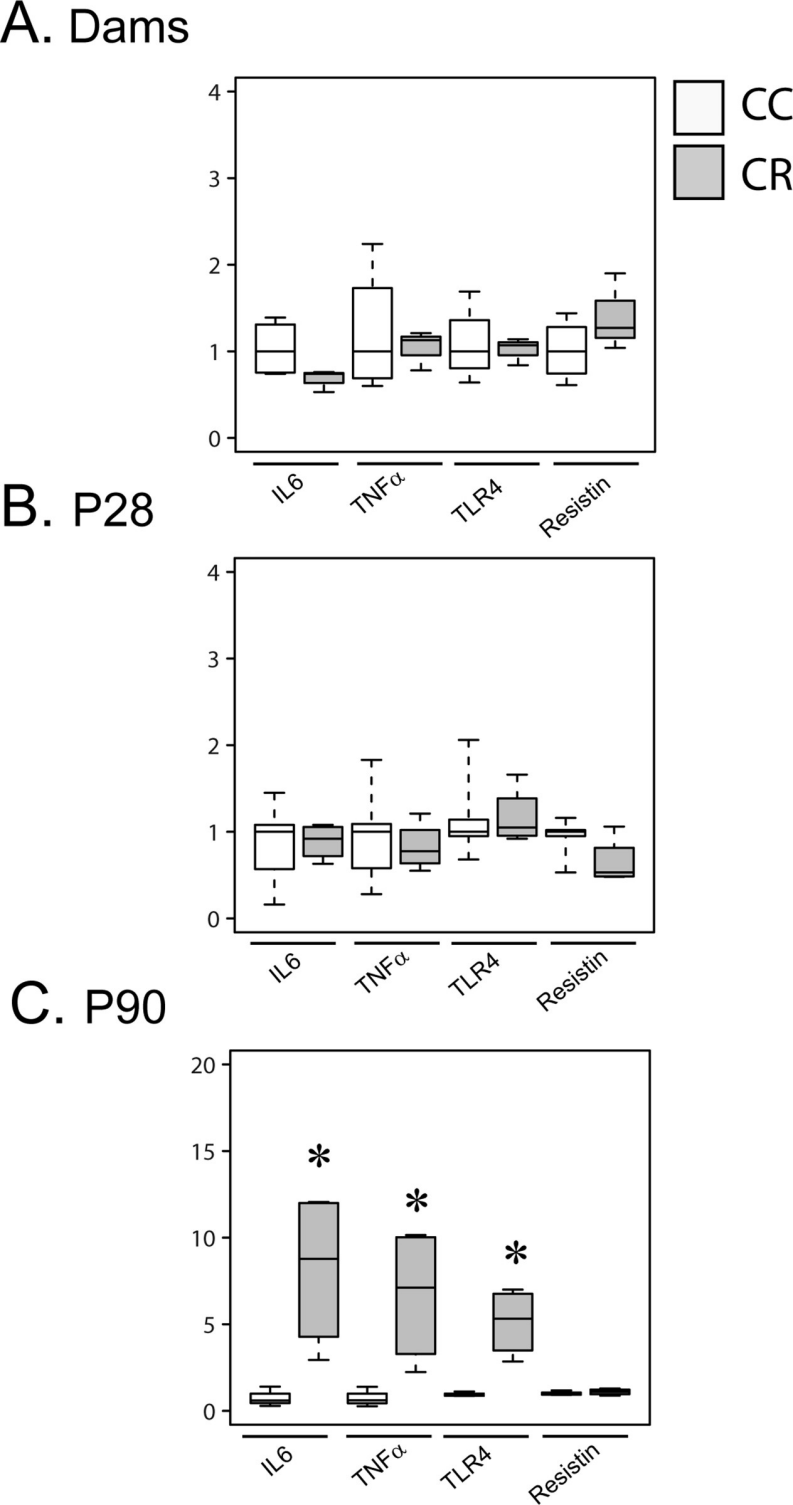
Maternal resistin increases cortical inflammation markers of offspring at p90. Total mRNA prepared from the cortex of dams and offspring (p28 and p90) were used to determine gene expression of different markers using RT-qPCR. Gene expression was normalized to GAPDH, 18S and Actin. Cortex inflammatory markers are presented in panel A for dams, panel B for p28 offspring and panel C for p90 offspring. Data are presented as boxplots (n = 4–5). *P<0.05 when comparing CC group to CR group using non-parametric Mann & Whitney statistical test.

To investigate whether maternal resistin treatment affects other brain regions, we have measured the expression of proinflammatory markers in the cortex. This treatment had no effect in dams and p28 offspring (Fig 4A and 4B). In p90 offspring, the cortex expression of IL6, TNFα and TLR4 are significantly higher in CR as compared to CC group, whereas resistin expression is not affected (Fig 4C).

### Increased expression of IL6 and TNFα in the adipose tissue of offspring born to dams treated with resistin

To determine whether maternal resistin has also impacted peripheral tissues we measured the expression of proinflammatory markers in adipose tissue, liver and muscle.

**Fig 5:**
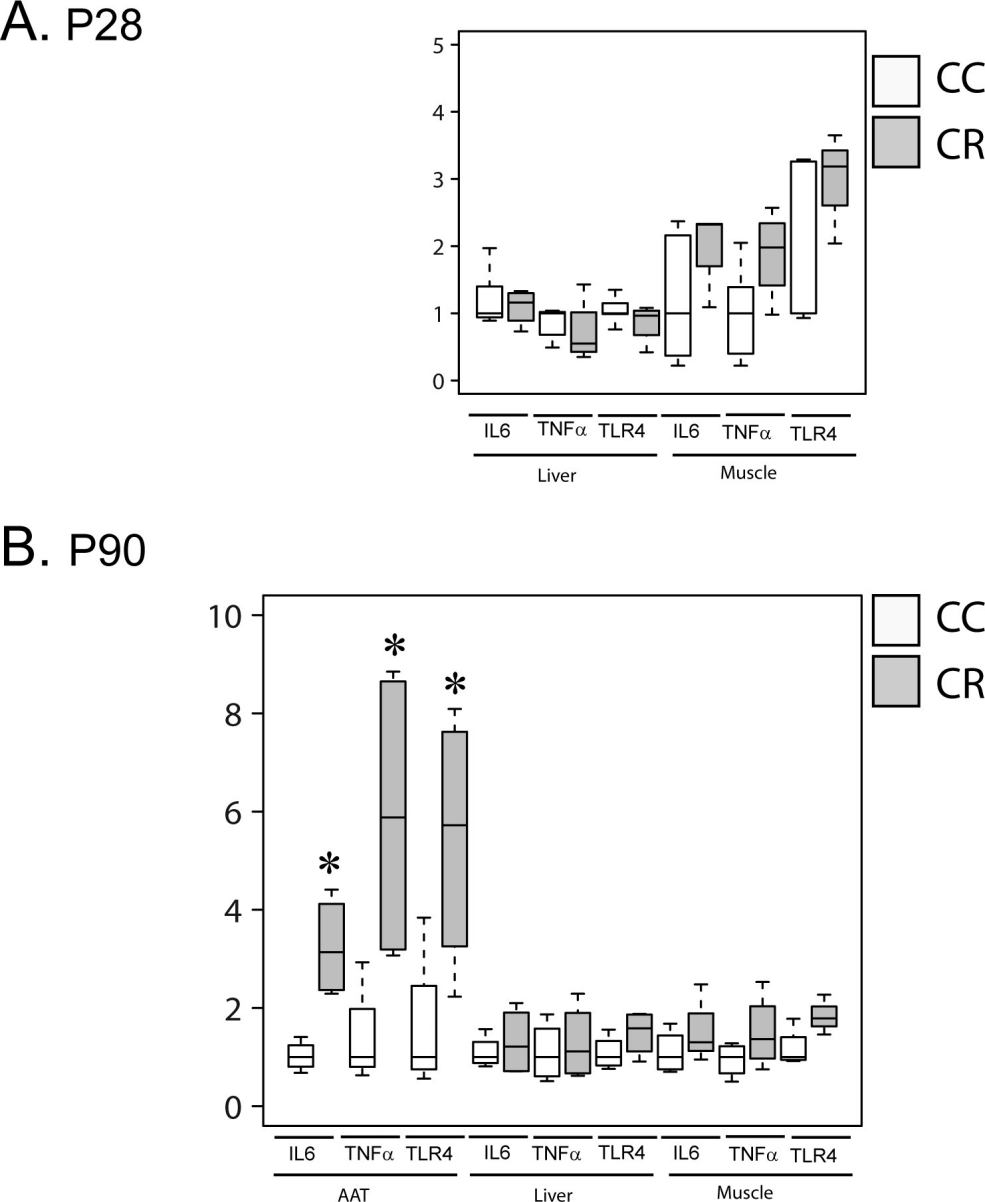
Maternal resistin increases adipose tissue inflammation markers of P90 offspring. Total mRNA prepared from abdominal adipose tissue (AAT), liver and muscle of p28 and p90 offspring were used to determine gene expression of inflammatory markers (IL6, TNFα and TLR4) using RT-qPCR. Gene expression was normalized to GAPDH, actin and 18S. Panel A presents inflammatory markers in liver and muscle of p28 offspring. Panel B presents inflammatory markers of AAT, liver and muscle of p90 offspring. Results are presented as boxplots (n = 4–5). *P<0.05 when comparing CC group to CR group using non-parametric Mann & Whitney statistical test.

At p28, resistin treatment did not affect inflammatory markers in liver and muscle (Fig 5A). At p28, the adipose tissue was not studied due to the very weak amount of adipose tissue at this age.

At p90, we measured the expression of IL6 and TNFα in offspring born to dams treated or not with resistin. We show as in the hypothalamus that CR group exhibits significantly higher expression levels of IL6, TNFα and TLR4 in adipose tissue as compared to CC group (Fig 5B). In contrast, in liver and muscle the expression of these inflammatory markers was not affected (Fig 5B).

## Discussion

During pregnancy, maternal obesity is associated with metabolic inflammation attributed to increased adipose tissue and circulating proinflammatory cytokine levels [1, 27]. In addition, maternal obesity modifies the secretion of adipokines including resistin that is known to be involved in the onset of inflammation [13]. Indeed, resistin is considered as a potential link between obesity and insulin resistance. However, the pro-inflammatory role of resistin during perinatal period is unknown. Furthermore, the correlation between circulating levels of resistin and the BMI of pregnant women are conflicting. Some of these studies revealed positive correlation between resistin plasma levels and the BMI in obese pregnant women and others showed similar plasma resistin levels between obese and non-obese women [22, 24, 28, 29]. However, there is a consensus concerning the deleterious effects of maternal inflammation, even though the specific effect of resistin has not been investigated independently of the controversy concerning its secretion by adipocyte in humans. Thus, the mouse model is of interest to investigate the effect of resistin in perinatal period. In this extensive preliminary study in the field, we investigated the long-term effects of maternal resistin on the predisposition of offspring to develop hypothalamic neuroinflammation and subsequent metabolic disorders. Here, we focused on the effects of resistin independently of systemic inflammation on normal dams and studied the long-term effects of this treatment on offspring and attempted to answer the question whether maternal resistin predisposes offspring to hypothalamic inflammation. For this purpose, the chosen resistin dose induced a slight but not significant increase of resistin plasma levels to avoid a high inflammation that could lead to abortion. However, resistin plasma levels of dams are higher when compared to offspring and this could be attributed to gender as previously reported [30]. We demonstrate that resitin treatment during pregnancy and lactation periods induced long-term effects on hypothalamic inflammation of male offspring associated with the alteration of metabolic parameters. Resistin treatment of dams did not modify their metabolic status or inflammatory markers as evidenced by unchanged glycemia, insulinemia and body weight. Furthermore, IL6 and TNFα plasma levels were not affected. Thus, in these conditions, we selectively investigated the impact of resistin perinatal treatment independently from systemic pro-inflammatory cytokine changes. At weaning (p28), offspring born to dams treated with resistin, CR group, exhibited lower resistin plasma levels without any changes in the other circulating parameters. This could be attributed to the alteration of adipose tissue resistin secretion consequently to perinatal resistin treatment that acts as a negative feedback regulatory loop, however the mechanism is unknown. This decrease is most likely compensated by the significant increase of TLR4, which is the binding site of resistin, and this could explain the increased expression hypothalamic inflammatory markers. Indeed, the expression of pro-inflammatory markers (IL6, NFκb, TNFα) was significantly increased in the hypothalamus of CR group as well as microgliosis markers (ICAM, CD68, IBA1) and astrogliosis (GFAP) markers. This suggests a potential hypothalamic neuroinflammation resulting from the maternal resistin treatment. Importantly, the long-term consequences of maternal resistin seem to differentially affect other brain regions such as the cortex. Indeed, inflammatory markers were not affected in the cortex of dams and p28 offspring, the effect is nevertheless observed in p90 CR group with increased IL6, TNFα and TLR4 expression. This indicates that the hypothalamus is affected earlier than the cortex. Additionally, p28 CR group exhibits a significant diminution of hypothalamic insulin and leptin receptor associated to the up-regulation of SOCS3 a negative regulator of insulin/leptin signaling. However, the expression level of PTP-1B was reduced in CR group as compared to CC group. These data show most likely the impairment of both IR and ObRb early in life. This finding suggests a strong impact later in life as previously described where leptin plays a crucial role in the neuronal organization of hypothalamic nuclei and especially ARC to PVN neuronal projections [31]. Indeed, the alteration of leptin action early in life has long-term impact on energy homeostasis [32, 33] and contributes to metabolic diseases. We have also investigated the impact of resistin perinatal treatment on the predisposition of offspring to inflammation and glucose intolerance when challenged with HFD. In fact, we have anticipated that using HFD will exacerbate the difference between CC and CR offspring and determine whether offspring born to dams treated with resistin are prone to inflammation and metabolic disorders. Indeed, HFD is known to promote these disorders. However, in future experiments It will be interesting to perform identical studies using Chow diet. At p90, CR group showed a significant increase in body weight gain and plasma insulin levels associated with slight glucose intolerance. Indeed, we have shown that AUC of GTT curves are significantly different between CC and CR, but non parametric ANOVA test showed no significant difference between the two groups when considering the time course curves. Thus, we conclude that maternal resistin led to a slight glucose intolerance, but when combined to the hyperinsulinemia found in CR group this fairly reveals most likely a potential insulin resistance. Plasma levels of IL6 and TNFα were not modified. However, as at p28, TLR4 was up-regulated in the hypothalamus despite a stable resistin plasma level this may contribute to hypothalamic inflammation. Indeed, hypothalamic expression of IL6, resistin, TLR4 and TNFα is increased in CR group at p90 as compared to CC group, revealing a sustained expression of inflammation markers in the hypothalamus. The expression levels of reactive astrogliosis and microgliosis markers were similar between CR and CC except for ICAM as well as IR and ObRb. This could be attributed to the fact that both groups were fed HFD and this most likely attenuated the differences except for the hypothalamic neuroinflammation markers. Our findings also show the up-regulation of IL6 and TNFα in the adipose tissue of CR group as compared to CC but not in liver or muscle. This indicates that the hypothalamus and adipose tissue of offspring are the most affected tissues in response to the augmentation of maternal resistin during gestation and lactation.

It is noteworthy to indicate that these extensive preliminary findings were obtained in male offspring and it is of great interest to extend these studies to female offspring in future investigations to determine whether long-term effects of maternal resistin concern both genders or if it is gender-dependent due to hormonal environment. Indeed, it has been recently demonstrated that neonatal overnutrition differentially affects males and females [34].

In conclusion, our findings demonstrate, for the first time to our knowledge, that maternal resistin predisposes male offspring to hypothalamic neuroinflammation and adipose tissue inflammation as evidenced by the up-regulation of inflammation markers independently from systemic inflammation markers associated to body weight gain, hyperinsulinemia and hyperleptinemia.
